# Analysis of the Most Viewed First 50 Videos on YouTube about Breast Cancer

**DOI:** 10.1155/2020/2750148

**Published:** 2020-05-25

**Authors:** Isil Yurdaisik

**Affiliations:** Department of Radiology, Istinye University Medical Park Gaziosmanpasa Hospital, Istanbul, Turkey

## Abstract

**Objective:**

Breast cancer is the most common cancer type among women worldwide. Today, health consumers search the Internet to gain health information about many diseases including breast cancer. YouTube™ is the second most commonly used website on the Internet. However, the quality and accuracy of health-related YouTube™ videos are controversial. The objective of this study was to investigate the quality and accuracy of breast cancer-related videos on YouTube™. *Material and Methods*. “Breast cancer” keyword was entered into YouTube™ search bar, and after excluding advertisement, duplicate, and non-English videos, the first most viewed 50 videos were analyzed. Videos' length, the number of views, comments, likes, and dislikes were recorded. DISCERN and JAMA scores and Video Power Index (VPI) values of the videos were calculated. All videos were evaluated by two independent radiologists experienced on breast cancer. The correlation between the two observers was also analyzed.

**Results:**

Of all videos, 14% were uploaded by physicians, 26% by health channels, 20% by patients, 10% by news channels, 2% by herbalists, 2% by blog channels, and 2% by nonprofit activism channels. The mean DISCERN score was calculated as 26.70 ± 10.99 and the mean JAMA score as 2.23 ± 0.97. The mean VPI value, which was calculated to determine the popularity of the videos, was found as 94.10 ± 4.48. A strong statistically significant correlation was found between the two observers in terms of both DISCERN and JAMA scores. There was an excellent agreement between the two observers.

**Conclusion:**

The overall quality of the viewed videos was found as poor. Healthcare professionals should be encouraged to upload breast cancer-related videos with accurate information to promote patients for screening and direct them appropriately.

## 1. Introduction

Breast cancer is the most common type of cancer among women worldwide. In 2018, over 2 million new breast cancer cases were found with an incidence slightly lower than lung cancer [1]. Breast cancer often leads to an increased economic burden on affected women, their families, and society. However, early diagnosis and treatment are associated with a reduction in negative outcomes caused by breast cancer. For this purpose, guiding people with accurate information on breast cancer plays an important role in the solution of this problem.

Today, the Internet is one of the most commonly used sources in order to access health information. YouTube™ is one of the Internet platforms used for this purpose. YouTube™, which was introduced for the first time in 2005, is the second most commonly visited website all over the world. Since that time, YouTube™ has become an increasingly important medium on which health information is shared between healthcare consumers and professionals [2]. The number of videos viewed on YouTube™ is 5 million daily and 300 hours of videos are uploaded to YouTube per minute [3].

Studies have shown that the Internet is one of the leading information sources for patients who have concerns about their diseases [4]. Cancer patients and their caregivers seek information about the management and prognosis of the disease and therapeutic alternatives [5]. In a study, 92% of cancer patients reported the Internet as a source empowering them in making decisions for treatment [6]. In a study by Yakren et al. on 223 cancer patients, 44% of the patients were found to use the Internet for gaining cancer-related information [7]. The rates of Internet usage among patients with breast cancer have been reported between 42% and 49% [8, 9]. However, it is known that the majority of these patients do not discuss the information, which they obtain from the Internet, with their physicians.

With the widespread use of the Internet, everyone has easy access to health information. However, assessment of the quality and scientific accuracy of this information is usually not possible for laypersons. While the distribution of health information to such a wide audience can provide a valuable opportunity, misleading and even harmful unfiltered content can be harmful. Studies have reported that in general health information on the Internet is of low quality. On the other hand, providing high-quality health information via the Internet is necessary in order to establish strong public health systems [10]. Therefore, quality and scientific accuracy of health-related information on commonly used social media platforms such as YouTube should be evaluated by healthcare professionals and accordingly, policies should be developed in order to prevent the uploading of misleading content.

In this study, we aimed at investigating the quality and scientific accuracy of the most viewed first 50 videos on YouTube™ that were accessed by using the “breast cancer” keyword.

## 2. Material and Methods

“Breast Cancer” keyword was entered into the search bar, “the most viewed” option was chosen among the search options provided by YouTube™ and the results were evaluated. Advertisements, duplicate videos, and non-English videos were excluded, and the remaining 50 videos were included in the analysis. Whether videos have real or animation content, uploaders, video content, video length, and the number of views, comments, likes, and dislikes were recorded. In order to assess the popularity of the videos, Video Power Index (VPI) values were calculated using the following formula:

(1) $$\begin{array}{c} \text{VPI}=\text{number of likes}/\left(\text{number of likes}+\text{number of dislikes}\right)\times 100. \end{array}$$

In order to avoid bias that may be resulted from the upload date of the video on YouTube™, the mean daily view number of the videos was calculated according to the following formula: [total view count determined during viewing of the video by the observers date of viewing the video by the observers–upload date of the video to YouTube™(days)].

Quality and scientific accuracy of the videos were evaluated by two independent experienced radiologists (Observer 1: 25-year experienced Asst. Prof., Observer 2: 8-year specialist). The evaluation was made using DISCERN (Quality Criteria for Consumer Health Information) and JAMA (Journal of the American Medical Association) scoring systems. In order to avoid bias, DISCERN and JAMA scores were evaluated separately by the observers. The mean DISCERN and JAMA scores were calculated by averaging individual DISCERN and JAMA scores of observers.

### 2.1. DISCERN Scoring System

DISCERN scoring is a tool used to assess the quality of videos uploaded to YouTube™. DISCERN scale consists of 15 questions about health-related content. The observers evaluate the content via a 5-point scale, and the total score varies between 15 and 75 points. DISCERN scores are classified as “excellent” between 63-75 points, “good” between 51-62 points, “average” between 39-50 points, “poor” between 28-38 points, and “very poor” for <28 points (Table 1) [11].

### 2.2. JAMA Scoring System

JAMA scoring system is a quality scale used for the evaluation of website contents including health information. This scale consists of 4 subscales as “Autorship, Attribution, Disclosure and Currency”. Each item is evaluated with 0 (does not meet the requested criterion) or 1 (meets the requested criterion) point. Minimum 0 and maximum 4 points can be obtained from the scale (Table 2) [12].

### 2.3. Statistical Analysis

Data obtained in the study were analyzed using SPSS 20.0 statistical package software. The normality of the variables was analyzed with the Kolmogorov-Smirnov test. Continuous variables are expressed as mean ± standard deviation, and median (minimum-maximum), while categorical variables are expressed by number (*n*) and percentage (%). The comparison of DISCERN and JAMA scores between physician and nonphysician video uploaders was performed with Mann-Whitney *U* test. The correlation between DISCERN and JAMA scores of the observers was examined with Spearman's correlation analysis. *P* < 0.05 values were considered statistically significant.

## 3. Results

**Figure 1:**
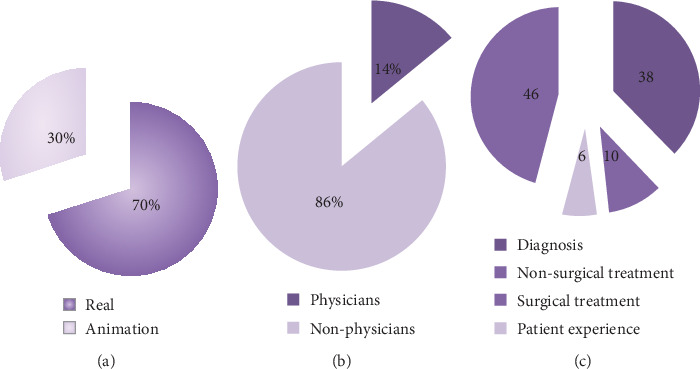
(a) Rate of the real and animation videos. (b) Rate of the videos uploaded by physicians and nonphysicians. (c) Distribution of the videos by general contents.

**Figure 2:**
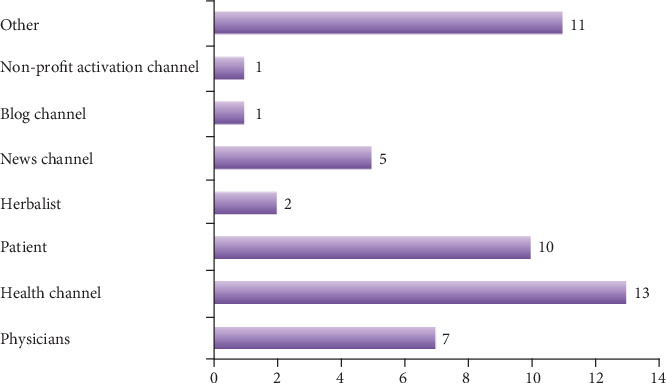
Distribution of the videos by uploaders.

When the general contents of the examined videos were evaluated; 46% (*n* = 23) included patient experience, 38% (*n* = 19) diagnosis, 10 (*n* = 5) nonsurgical treatment, and 6% (*n* = 3) surgical treatment content. Of all videos, 35% included real and 15% animated images. Of the videos, 14% were uploaded by physicians, 26% by health channels, 20% by patients, 10% by news channels, 2% by herbalists, 2% by blog channels, and 2% by nonprofit activism channels. General features of the videos are given in Figures 1(a)–1(c). The distribution of the videos by uploaders is seen in Figure 2. The numbers of comments, likes, and dislikes of the videos are shown in Table 3.

The main characteristics of the viewed videos including length, number of views, time from the date of upload, mean number of daily views, number of comments, number of likes, number of dislikes, and Video Power Index (VPI) values are given in Table 4.

The mean DISCERN score was calculated as 26.70 ± 10.99 and the mean JAMA score as 2.23 ± 0.97. DISCERN and JAMA scores of the first and second observers are given in Table 5.

When the quality of the videos was evaluated according to DISCERN scores; the quality was found as very poor in 66% (*n* = 33), poor in 20% (*n* = 10), average in 12% (*n* = 6), and excellent in 2% (*n* = 1) of the videos. The only video evaluated as excellent was uploaded by a physician.

**Figure 3:**
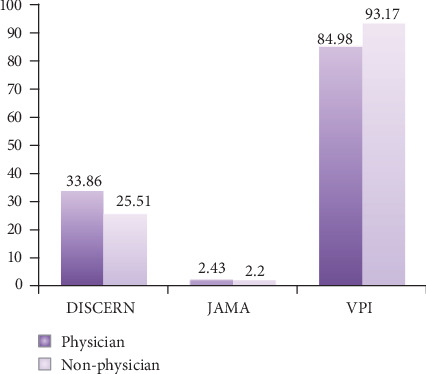
DISCERN, JAMA, and VPI scores of the videos uploaded by physicians and nonphysicians.

When the videos uploaded by physicians and nonphysicians were evaluated in terms of DISCERN, JAMA, and VPI scores; the mean DISCERN score was found as 33.86 ± 11.26 in the videos uploaded by physicians and 25.51 ± 10.99 in the videos uploaded by nonphysicians. Accordingly, the mean DISCERN score of the videos uploaded by physicians was statistically significantly higher than the mean DISCERN score of the videos uploaded by nonphysicians (*P* = 0.001). No statistically significant difference was found between the videos uploaded by physicians and nonphysicians in terms of JAMA scores and VPI values (*P* > 0.05) (Figure 3). In addition, no statistically significant difference was found between the animated and real videos in terös of DISCERN, JAMA, and VPI values (*P* > 0.05).

When DISCERN and JAMA scores of the observers were examined by the correlation analysis; a strong statistically significant correlation was found between the two observers in terms of both DISCERN and JAMA scores (Table 6). Accordingly, there was an excellent agreement between the two observers.

## 4. Discussion

To our knowledge, this study is the first in the literature to investigate the quality and scientific accuracy of the most viewed videos about breast cancer on YouTube™. Given the high prevalence of breast cancer worldwide, a high portion of patients are expected to seek information about the diagnosis, prognosis, and treatment of the disease. The Internet is among the leading sources of information that are used by patients for this purpose. Eight of each ten patients consult the web for searching health-related information [13]. Once the diagnosis of cancer is made, 71% of patients search the Internet in order to gain more information [14]. However, quality and scientific accuracy of health information uploaded to the platforms such as YouTube™ are controversial. When consumers obtain information from YouTube™ for making decisions about health, three major concerns have been determined: (1) YouTube™ is used as a medium promoting unscientific treatments that are yet to be approved by an appropriate authority [15], (2) YouTube™ has information with contradicting reference standards/guidelines, and (3) YouTube™ has a potential to change the belief of patients about controversial issues. Social media has the potential to aid in closing the gap in health literacy. However, despite this opportunity, there is likelihood for dissemination of inaccurate and even harmful information at the same time.

Professionals from various medical disciplines and fields should understand how their patients tend to use misleading and limited information sources.

In the present study, the overall quality of the viewed videos was poor. We think that the low rate of videos uploaded by physicians might have played a role in this result. In our study, only 14% of the videos were uploaded by physicians. Studies in the literature have reported different rates. In a study by Gokcen et al., evaluating YouTube™ videos about disc herniation, 48% of the videos were uploaded by physicians [16]. However, similar to our study, some studies have reported lower rates [17]. The difference between the rates of videos uploaded by physicians that have been reported by different studies might be caused by the topic searched. As a topic of search, breast cancer included information variability in a wide spectrum, and we think this increases the likelihood of videos uploaded by laypersons.

In the literature, there are various scoring systems used to assess the quality and scientific accuracy of videos on the Internet [18, 19]. In our study, we used DISCERN and JAMA scoring systems that have been commonly used in previous studies. In our study, the mean DISCERN score was significantly higher in the videos uploaded by physicians compared to the videos uploaded by nonphysicians, whereas no statistically significant difference was found between the videos uploaded by physicians and nonphysicians in terms of the mean JAMA and VPI scores. On the other hand, the mean number of likes was higher in the videos uploaded by nonphysicians than the videos uploaded by physicians. Although studies have reported that videos uploaded by physicians are of a higher quality, it has been stated that the number of views and likes may be lower since these videos may not be understood by patients [20, 21]. In our study, DISCERN scores of the videos uploaded by physicians were higher than those uploaded by nonphysicians, although the quality of the videos uploaded by physicians was insufficient.

In our study, the rate of videos uploaded by patients was found as 20%. Other studies investigating cancer-related videos have reported similar results [22]. In our study, the mean DISCERN score was 22.60 and the mean JAMA score was 2 in these videos, lower than the overall mean value. In addition, the quality of these videos was evaluated as poor. On the other hand, the mean VPI value was higher in the videos uploaded by patients compared to the overall mean value. This has been attributed to that anecdotal health information that is seen as important by patients [23]. Videos uploaded by patients that include their experience are likely to mislead, to affect treatment decisions, and to cause problems in the relationship between patients and physicians. The most common content of the videos was found as followed by patient experience. However, these videos did not include information about breast ultrasound, mammography, breast magnetic resonance imaging, and breast biopsy that are used for diagnosis, screening, and follow-up in breast cancer. Even the most basic information about in which age group ultrasonography and/or mammography should be performed for screening purposes was absent in the viewed videos. There was no video including information for patients who have hesitancy for mammography. While the importance of early diagnosis in breast cancer is continuously highlighted, unfortunately, these videos did not include information about screening methods that are crucial in early diagnosis.

In our study, 70% of the videos included real and 30% animated images. Animation-containing videos were mostly uploaded by health channels. It has been proposed that animation-containing videos are found more useful by patients, and this promotes the production of more animated videos [24]. However, in our study, no statistically significant difference was found between the animated and real images in terms of DISCERN, JAMA, or VPI scores (*P* > 0.05).

In the literature screening, there was no study directly investigating YouTube™ videos about breast cancer. Nevertheless, there were studies investigating the quality and validity of the Internet videos about several cancer types including colorectal and prostate cancers. In a study by Sahin et al. on YouTube™ videos as an information source about colorectal cancer, the need for more comprehensive videos that will be uploaded by more professional persons and can be easily identified by patients was emphasized. The authors concluded that currently, YouTube™ may not be an educational source suitable for each patient with colorectal cancer [25]. Again, in a study Corey et al., which analyzed contents of YouTube™ videos about prostate cancer, concerns were stated about the accuracy of prostate cancer contents provided by YouTube™ videos [26]. In a study by Steinberg et al., video quality was found as fair or poor in 73% of YouTube™ videos about prostate cancer [27]. In our study, video quality was found as very poor or poor in 43 of the most viewed first 50 videos about breast cancer on YouTube™. Given the high incidence of breast cancer, these results supported the concerns about the quality and accuracy of health content on YouTube™.

Therefore, policy makers should develop policies, strategies, and regulations in order to prevent the uploading of misleading content on YouTube and similar platforms based on the opinions of health professionals. In addition, healthcare professionals should be encouraged to upload accurate content and guide patients to access accurate and appropriate sources of health-related information.

This study has some limitations. First, only the most viewed 50 videos identified with search results were included. However, studies have reported that people who search the Internet usually focus on the first results [28, 29]. Second, continuous change of YouTube™ videos might make our instant search a limitation. Further studies with a higher number of videos that will analyze video comments in more detail and in longer periods are needed.

In conclusion, this study fills a gap in the literature about an important issue. YouTube™ provides an easily accessible platform for health consumers to obtain information on the screening, diagnosis, prognosis, and treatment of their disease. However, as indicated by our results, quality and scientific accuracy of breast cancer-related YouTube™ videos were found to be insufficient. It is important for healthcare professionals to be aware of the video content on YouTube™ which is used by their patients. For this purpose, healthcare professionals should be encouraged for uploading videos with accurate information that will appropriately direct patients for screening and treatment.

## Figures and Tables

**Table 1:** DISCERN scoring system.

DISCERN scoring system						
Section	Questions	No	Partly			Yes
Reliability of the publication	(1) Explicit aims	1	2	3	4	5
	(2) Aims achieved	1	2	3	4	5
	(3) Relevance to patients	1	2	3	4	5
	(4) Source of information	1	2	3	4	5
	(5) Currency (date) of information	1	2	3	4	5
	(6) Bias and balance	1	2	3	4	5
	(7) Additional sources of information	1	2	3	4	5
	(8) Reference to areas of uncertainty	1	2	3	4	5
Quality of information on treatment choices	(9) How treatment works	1	2	3	4	5
	(10) Benefits of treatment	1	2	3	4	5
	(11) Risks of treatment	1	2	3	4	5
	(12) No treatment options	1	2	3	4	5
	(13) Quality of life	1	2	3	4	5
	(14) Other treatment options	1	2	3	4	5
	(15) Shared decision making	1	2	3	4	5

**Table 2:** JAMA scoring system.

JAMA scoring system		Rating	
Section		No	Yes
Authorship	Authors and contributors, their affiliations, and relevant credentials should be provided	0	1
Attribution	References and sources for all content should be listed clearly, and all relevant copyright information should be noted	0	1
Disclosure	Website “ownership” should be prominently and fully disclosed, as should any sponsorship, advertising, underwriting, commercial funding arrangements or support, or potential conflicts of interest	0	1
Currency	Dates when content was posted and updated should be indicated	0	1

**Table 3:** Distribution of the videos' numbers of comments, likes, and dislikes.

	Number of videos	Comments	Likes	Dislikes
Physicians	7	144	1937	427
Health channel	13	282	2453	270
Patient	10	300	2031	82
Herbalist	2	310	3500	134
News channel	5	1892	12960	726
Other	13	675	9015	406

**Table 4:** Distribution of the videos' numbers of comments, likes, and dislikes.

Variables	Mean ± standard deviation	Median
		(Minimum-maximum)
Video length	7.63 ± 6.50	4.47 (1.28-60.07)
Number of view	1,114,268 ± 1,458,356	412,01 (133,356-7,590,223)
Time from the date of upload	1,549.54 ± 978.22	1,446.5 (110-3,903)
Number of daily views	1,036.96 ± 1,431.93	461,21 (36.78-6,830.95)
Number of comments	535 ± 1,076.74	198 (0-6,658)
Number of likes	5,214 ± 7,298.41	2,750 (0-30,000)
Number of dislikes	336.23 ± 469.27	109 (1-2,000)
Video Power Index (VPI)	94.10 ± 4.48	93.90 (82.89-99.15)

**Table 5:** DISCERN and JAMA scores of the first and second observers.

Variables	Mean ± standard deviation	Median
		(Minimum-maximum)
DISCERN score (observer 1)	26.84 ± 10.92	25 (15-69)
JAMA score (observer 1)	2.24 ± 0.95	2 (1-4)
DISCERN score (observer 2)	26.56 ± 11.05	25 (15-67)
JAMA score (observer 2)	2.22 ± 0.99	2 (1-4)
Mean DISCERN score of the observers	26.70 ± 10.99	25 (15-69)
Mean JAMA score of the observers	2.23 ± 0.97	2 (1-4)

**Table 6:** Correlation analysis of DISCERN and JAMA scores between the two observers.

	Mean ± SD	Median	*r* ^∗^, *P*	Cronbach *α*
		(Min-max)		
DISCERN 1	26.84 ± 10.92	25 (15-69)	0.976 *P* < 0.01	0.994
DISCERN 2	26.56 ± 11.05	25 (15-67)		
JAMA 1	2.24 ± 0.95	2 (1-4)	0.861 *P* < 0.01	0.926
JAMA 2	2.22 ± 0.99	2 (1-4)		

^∗^Spearman *r* correlation coefficient.

## Data Availability

The [Health content scoring systems] data used to support the findings of this study are included within the article.
